# Predicting Survival within the Lung Cancer Histopathological Hierarchy Using a Multi-Scale Genomic Model of Development

**DOI:** 10.1371/journal.pmed.0030232

**Published:** 2006-07-04

**Authors:** Hongye Liu, Alvin T Kho, Isaac S Kohane, Yao Sun

**Affiliations:** **1**Children's Hospital Informatics Program, Children's Hospital Boston, Boston, Massachusetts, United States of America; **2**Department of Newborn Medicine, Children's Hospital Boston, Boston, Massachusetts, United States of America; **3**Harvard–Massachusetts Institute of Technology, Division of Health Sciences and Technology, Cambridge, Massachusetts, United States of America; Ludwig Maximilians Universität MünchenGermany

## Abstract

**Background:**

The histopathologic heterogeneity of lung cancer remains a significant confounding factor in its diagnosis and prognosis—spurring numerous recent efforts to find a molecular classification of the disease that has clinical relevance.

**Methods and Findings:**

Molecular profiles of tumors from 186 patients representing four different lung cancer subtypes (and 17 normal lung tissue samples) were compared with a mouse lung development model using principal component analysis in both temporal and genomic domains. An algorithm for the classification of lung cancers using a multi-scale developmental framework was developed. Kaplan–Meier survival analysis was conducted for lung adenocarcinoma patient subgroups identified via their developmental association. We found multi-scale genomic similarities between four human lung cancer subtypes and the developing mouse lung that are prognostically meaningful. Significant association was observed between the localization of human lung cancer cases along the principal mouse lung development trajectory and the corresponding patient survival rate at three distinct levels of classical histopathologic resolution: among different lung cancer subtypes, among patients within the adenocarcinoma subtype, and within the stage I adenocarcinoma subclass. The earlier the genomic association between a human tumor profile and the mouse lung development sequence, the poorer the patient's prognosis. Furthermore, decomposing this principal lung development trajectory identified a gene set that was significantly enriched for pyrimidine metabolism and cell-adhesion functions specific to lung development and oncogenesis.

**Conclusions:**

From a multi-scale disease modeling perspective, the molecular dynamics of murine lung development provide an effective framework that is not only data driven but also informed by the biology of development for elucidating the mechanisms of human lung cancer biology and its clinical outcome.

## Editors' Summary

**Background.:**

Lung cancer causes the most deaths from cancer worldwide—around a quarter of all cancer deaths—and the number of deaths is rising each year. There are a number of different types of the disease, whose names come from early descriptions of the cancer cells when seen under the microscope: carcinoid, small cell, and non–small cell, which make up 2%, 13%, and 86% of lung cancers, respectively. To make things more complicated, each of these cancer types can be subdivided further. It is important to distinguish the different types of cancer because they differ in their rates of growth and how they respond to treatment; for example, small cell lung cancer is the most rapidly progressing type of lung cancer. But although these current classifications of cancers are useful, researchers believe that if the underlying molecular changes in these cancers could be discovered then a more accurate way of classifying cancers, and hence predicting outcome and response to treatment, might be possible.

**Why Was This Study Done?:**

Previous work has suggested that some cancers come from very immature cells, that is, cells that are present in the early stages of an animal's development from an embryo in the womb to an adult animal. Many animals have been closely studied so as to understand how they develop; the best studied model that is also relevant to human disease is the mouse, and researchers have previously studied lung development in mice in detail. This group of researchers wanted to see if there was any relation between the activity (known as expression) of mouse genes during the development of the lung and the expression of genes in human lung cancers, particularly whether they could use gene expression to try to predict the outcome of lung cancer in patients.

**What Did the Researchers Do and Find?:**

They compared the gene expression in lung cancer samples from 186 patients with four different types of lung cancer (and in 17 normal lung tissue samples) to the gene expression found in normal mice during development. They found similarities between expression patterns in the lung cancer subtypes and the developing mouse lung, and that these similarities explain some of the different outcomes for the patients. In general, they found that when the gene expression in the human cancer was similar to that of very immature mouse lung cells, patients had a poor prognosis. When the gene expression in the human cancer was more similar to mature mouse lung cells, the prognosis was better. However, the researchers found that carcinoid tumors had rather different expression profiles compared to the other tumors.

The researchers were also able to discover some specific gene types that seemed to have particularly strong associations between mouse development and the human cancers. Two of these gene types were ones that are involved in building and breaking down DNA itself, and ones involved in how cells stick together. This latter group of genes is thought to be involved in how cancers spread.

**What Do These Findings Mean?:**

These results provide a new way of thinking about how to classify lung cancers, and also point to a few groups of genes that may be particularly important in the development of the tumor. However, before these results are used in any clinical assessment, further work will need to be done to work out whether they are true for other groups of patients.

**Additional Information.:**

Please access these Web sites via the online version of this summary at http://dx.doi.org/10.1371/journal.pmed.0030232.

• MedlinePlus has information from the United States National Library of Medicine and other government agencies and health-related organizations [MedlinePlus]

• National Institute on Aging is also a good place to start looking for information [National Institute for Aging]

•  [The National Cancer Institute] and Lung Cancer Online [ Lung Cancer Online] have a wide range of information on lung cancer

## Abstract 3

Comparison of gene expression patterns in patients with lung cancer and in mouse lung development showed that those tumors associated with earlier mouse lung development had a poorer prognosis.

## Introduction

Lung cancer is the leading cause (28%) of cancer deaths worldwide [ 1, 2] and accounted for 163,500 new cases of cancer in the United States in 2004 [ 2]. Compared with the other major cancers—breast, colorectal, and prostate—it has seen only modest improvements in its survival rates and clinical outcome up to the present time, and is the only major cancer type to have increased in the number of deaths annually [ 2, 3].

Histopathologic heterogeneity is a major confounding factor in lung cancer diagnosis and treatment [ 4]. Classically, lung cancer comprises three primary histological subtypes: carcinoid, small cell, and non–small cell, which account for about 2%, 13%, and 86% of lung cancers, respectively. Non–small cell lung cancer (NSCLC) is further subdivided into at least three histologic subtypes: adenocarcinoma (AD), squamous cell carcinoma (SQ)/epidermoid, and large cell carcinoma. Small cell lung cancer (SCLC), the most aggressive form of lung cancer, includes small cell carcinoma, mixed small cell/large cell carcinoma, and combined small cell carcinoma. Carcinoids (COIDs) form a distinct histologic tumor subtype that may secrete bioactive molecules, and are further subdivided into typical and atypical varieties [ 5]. Tumors such as adenosquamous and neuroendocrine carcinomas possess histological characteristics of more than one subtype, whereas tumors from the same histopathologic subtype may have dissimilar clinical outcomes such as drug response [ 6, 7]. The differential histopathology between lung cancer subtypes is not always obvious or objective, and proper classification is a critical component of pretreatment evaluation. For instance, cases of COIDs being misdiagnosed as SCLC are not uncommon, yet typical treatments for COIDs and SCLC are very different [ 8, 9]. This heterogeneity has motivated several efforts to classify lung cancers by their molecular profiles [ 10– 14].

In a majority of molecular classification studies, hierarchical clustering methods are used that, in some cases, uncover previously unidentified disease subsets within a classical histopathologic subtype that have differential clinical outcomes. However, even though the molecular profiles of samples from one cancer subtype tend to occupy a common dendrogram cluster, there is significant admixing with samples from different cancer subtypes. Cluster configurations resulting from hierarchical algorithms are generally not well defined since they vary depending upon the input order of the same data [ 15, 16] and are often irreproducible [ 17]. More importantly, hierarchical clustering (or any purely correlative technique) alone does not provide a rational biological basis for disease classification.

It has been hypothesized that lung and other solid cancers arise from self-renewing progenitor cells that are capable of generating morphologically and functionally diverse progeny. An important corollary is the idea that embryonic development programs play key roles in tumor genesis and progression. The developmental–stem cell association for non-solid cancers such as leukemia is well established [ 18], but the case for solid tumors is less clear. Borczuk et al. [ 12] found that murine orthologs of marker genes for human AD and large cell lung carcinoma are associated with distinct stages of mouse lung development and biological function. AD markers were expressed late in mouse lung development, and were largely associated with differentiation and signal transduction. Large cell lung carcinoma markers were expressed earlier in mouse lung development, and were mainly involved in cell cycle and proliferation. Kho et al. [ 19] reported that in the central nervous system, primary human cancers showed molecular association with cognate organogenesis both temporally and genomically.

In contrast to previous molecular classification studies using hierarchical clustering on lung cancer data with no direct reference to development [ 10, 12– 14], we used cognate organ development as the primary foundation for a classification scheme and examined its clinical relevance. We considered the molecular associations of four human lung cancer subtypes to a developing mouse lung sequence and investigated the link between those associations and clinical outcome.

## Methods

### RNA Microarray Dataset of Mouse Lung Development and Human Lung Normal and Cancer Subtypes

The mouse lung development RNA data were derived from perfused, whole, wild-type mouse lung at ten distinct developmental days assayed on Affymetrix ( http://www.affymetrix.com) Mu11K microarrays [ 20]. These data are publicly available at http://lungtranscriptome.bwh.harvard.edu. The normal human lung (NL) tissue and cancer subtype RNA profiles were assayed on Affymetrix U95A microarrays; the corresponding clinical parameters for human patients have also been described [ 10] and are publicly available at http://research.dfci.harvard.edu/meyersonlab/lungca. We evaluated NL tissue ( *n =* 17) and four lung cancer subtypes: AD ( *n =* 139; 125 with survival information), SQ ( *n =* 21), SCLC ( *n =* 6), and COIDs ( *n =* 20).

The human lung AD dataset used for independent validation of the findings is the one published in Beer et al. [ 21]. The raw microarray data and other associated data such as patient survival and tumor stage are publicly available at http://dc.nci.nih.gov/dataSets. The dataset includes 86 lung ADs and ten NL samples assayed with Affymetrix Hu6800 microarrays.

### Mouse–Human Orthologous Gene Pairs and Genomic Vectors

The sequence homology mapping between mouse and human genes—as identified by their Entrez GeneID accession numbers—can be found on the NCBI HomoloGene Web site ( http://www.ncbi.nlm.nih.gov/entrez/query.fcgi?db=homologene) [ 22]. Both the Mu11K and U95A microarray platforms have instances where more than one probeset on a platform possess a common Entrez GeneID, i.e., different probesets are assaying the same RNA transcript. In this instance, one probeset was selected to represent the Entrez GeneID/gene based on the number of Affymetrix present calls and the maximal coefficient of variation of the probeset's reported signal across the mouse (or human, for the human dataset) dataset. Between the mouse Mu11K and the human U95A platforms, 3,590 homologous ortholog gene pairs were found following this rule. Each mouse or human sample is represented as a 3,590-feature vector of expression measurements. The *j*th component in the mouse and human vectors are gene orthologs of one another. For example, if the 1,685th component of the mouse vector is the expression for the mouse gene *brain acyl-CoA hydrolase (Bach),* then the 1,685th component of the human vector is the expression of its human ortholog, *BACH.* For the independent validation with the separate human lung AD dataset, we found and thereby used 2,653 homologous genes common to the dataset mentioned above.

### Temporal and Genomic Principal Component Analysis of the Mouse Lung Development Data, and the Projection of Human Cancer Profiles into Mouse Genomic Framework/Space

Before applying principal component analysis (PCA) on the temporal axis of the mouse lung development dataset of 3,590 genes × ten stages, each gene's profile/signal across the ten lung stages was standardized to mean zero and variance one [ 19, 23]. The first three temporal principal components (PCs) captured 59.77% of the total temporal variance of the mouse lung development dataset.

In the PCA of the genomic axis (using all 3,590 genes) of the mouse lung development data, the first three genomic PCs captured 59.56% of the total genomic variance. The projection of human lung sample profiles into this genomic mouse lung development framework/space was done via the transformation where Φ′ _matrix of PCs_ x _old_vector_ = x _new_vector_ is applied [ 19]. Note that temporal and genomic PCAs—and subsequent projections of human profiles into the latter genomic space—were done separately for several different sets of mouse genes that were the outputs of the “top union” method (described below) to find a minimal subtype-discriminating gene set.

### Rank Normalization and Wilcoxon's Rank Sum Test for Differential Expression in Human Lung Samples

A nonparametric approach was used to determine genes that are differentially expressed between human lung cancer subtypes and NL samples. First, each human lung sample of 3,590 unique gene signals was rank-normalized. That is, each reported gene signal was replaced by a rank integer ranging from 1 to 3,590—depending upon the magnitude of its signal relative to the signals of the 3,589 other genes in that sample. Rank normalization renders the dataset less susceptible to the inherent “noise” of the measurement system across the different samples. Wilcoxon's rank sum test [ 24] was then applied to assess the likelihood (a *p*-value) of obtaining a Wilcoxon test statistic that is equal to or greater than the observed statistic between the cancer and normal groups, with the null hypothesis of no differential expression.

Genes were then ordered by their *p*-values. A Bonferroni correction factor set the *p*-value cut-off for statistical significance [ 25]. Any gene whose *p*-value fell below this cut-off was considered to be significantly differentially expressed between a cancer subtype and the NL, i.e., a top gene for that cancer subtype.

### A Top-Union Algorithm and Discrimination Measure for Determining Gene Sets That Best Discriminate between Human Lung Cancer Subtypes

A two-part top-union algorithm was designed to minimize “noise” in the mouse–human data, namely, to find a minimum set that discriminated the different lung cancer subtypes developmentally, based on gene signature in terms of cancer versus control.

The first part of the algorithm identified genes that are differentially expressed between each of the four human lung cancer subtypes with respect to NL tissue via Wilcoxon's test as described above. Four lists of genes were obtained, each 3,590 genes long and ordered by their Wilcoxon *p*-values. We found 405 top genes for AD, 737 for COID, 782 for SCLC, and 464 for SQ—the union of which are 1,148 unique genes. As a reality check, Figures S1– S4 show that applying the genomic PCA–projection method above to these respective gene subsets clearly discriminates each lung cancer subtype from the NL with respect to a mouse lung development background.

The second part of the algorithm used the union of the top gene sets (1,148 genes in this case) to construct a corresponding mouse lung development genomic framework and projected the human samples into the resulting space with the PCA–projection method above. We then quantified the discrimination of cancer subtypes in this genomic mouse lung development space (the standard Euclidean distance on PC1 and PC2) using a discrimination measure that is characterized by four parameters: ad_cc, the average distance between centroids of the closest pairs of cancer subtypes; *t,* the average tightness of cancer subtypes computed as the area of the convex hull of the human sample points divided by the number of samples in the subtype(the convex hull of a set of points is defined to be the smallest convex set C containing these points; a set C is convex if a line joining any given two elements in C is contained within C); ad_cn, the average distance between centroids of cancer subtypes and NL; and *n*_ovlp_, the number of overlapping sample points between different cancer subtypes—for each pair of distinct subtypes, we counted the number of samples located in the intersection of the corresponding two convex hulls.

For optimal discrimination between subtypes, the aim was to maximize ad_cc, ad_cn, and *t,* and minimize *n*_ovlp_. We defined the score _pca_ for this discrimination measure as the equal weighted sum of each score calculated from ad_cc, ad_cn, *t,* and *n*_ovlp_ alone. Score _pca_ is clearly a direct function of the genes that went into constructing the genomic development background.

Starting with the 1,148 top genes above, we sought a gene subset that maximizes score _pca_. The details of the procedure and pseudo codes are included in Protocol S1.

### Statistical Analysis

A nonparametric Pearson χ ^2^ test with Yates continuity correction was used to assess the significance of the mouse lung developmental profile segregation of genes that are significantly 2-fold up-regulated in human lung cancers versus genes that are significantly 2-fold down-regulated. The χ ^2^ tests were performed using R software ( http://www.r-project.org). All survival analyses were standard and performed using MedCalc software ( http://www.medcalc.be), and the statistical significance of the separation between the Kaplan–Meier (K-M) curves was evaluated using a log-rank (Mantel–Haenzel) test under the assumption of proportional hazards in the two groups being tested. Multiple-predictor comparisons were evaluated through Cox proportional-hazards regression. The Wilcoxon's rank sum test was carried out with Matlab (MathWorks; http://www.mathworks.com).

## Results

### Distinct Mouse Lung Development Profiles of Differentially Expressed Human Lung Cancer Genes

The mouse lung development expression profile of genes differentially expressed in human lung cancer with respect to NL tissue was examined. The lung development data are RNA profiles of perfused, whole, wild-type mouse lung at ten time points: embryonic days 12, 14, 16, and 18, and postnatal days 1, 4, 7, 10, 14, and 21 [ 26], covering the five main stages of mouse lung development [ 20, 27], measured on Affymetrix Mu11K microarrays. The human lung cancer RNA dataset has been described [ 10], and consists of 139 ADs, 21 SQs, six SCLCs, 20 lung COIDs, and 17 NL tissue samples—assayed on Affymetrix U95A microarrays. A total of 3,590 unique mouse–human orthologous gene pairs were found between the mouse and human RNA microarray platforms (see Methods).

**Figure 1:**
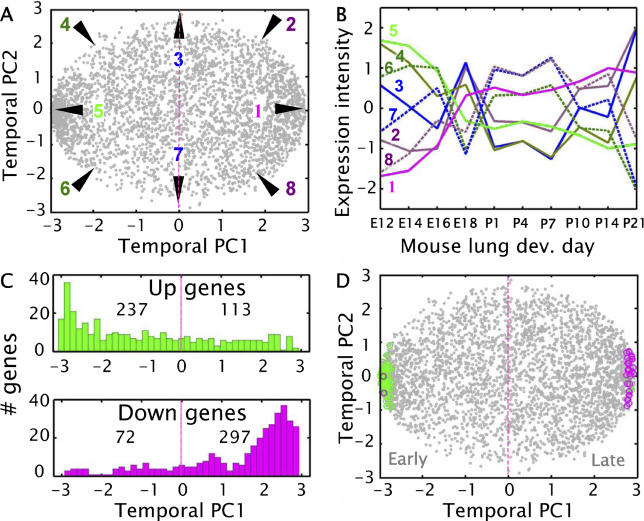
Mouse Lung Development Profiles in Temporal PC Representation, and the General Developmental Profile Segregation of Up- and Down-Regulated Genes in Human Lung Cancer (A) Expression profiles of all 3,590 unique genes during mouse lung development as represented in temporal PC1 and PC2. Each dot marks a gene. (B) Developmental profile examples of genes at the periphery of the disc-like scatter plot in (A) at 45° (π/4 radians starting at “3 o'clock”) rotational intervals. (C) Histograms of the mouse lung temporal PC1 coordinates of the 719 genes 2-fold significantly up- and down-regulated in any one of the four human lung cancer subtypes (χ ^2^ = 168.338, *p <* 0.001, OR = 8.652). (D) The profiles of the top 100 genes (68 cancer up-regulated [green circles] and 32 cancer down-regulated [magenta circles]) composing the malignancy signature (see Results) among all 3,590 mouse lung developmental gene profiles. Of the 68 cancer up-regulated genes, all but two are in the late developmental profile hemisphere. Of the 32 cancer down-regulated genes, all but two are in the early developmental profile hemisphere (χ ^2^ = 82.5185, *p <* 0.001, OR = 544).

In order to visualize the developmental profiles of 3,590 separate genes in a compact manner summarizing the large-scale developmental patterns, PCA [ 19, 23, 28] was applied on the temporal axis of the mouse lung development dataset of 3,590 mouse genes × ten developmental time points ( Figure 1). PCA [ 29] reduces the feature space dimensionality—i.e., features such as genes or samples—of a multivariate dataset and identifies the most variationally informative features (variationally informative features are the main contributors to global variation in the data matrix [ 30]). The original data are rewritten as an equivalent set of coefficients relative to a new basis of PCs. Each PC is a linear combination of the original features (here samples and time stages) and represents a direction of extremal variance in the feature space. The first PC (PC1) captures the greatest amount of total variance in the dataset. PC2 captures the next greatest contribution to variance. The disc-shaped scatter plot in Figure 1A shows the 3,590 genes rewritten with respect to the two most important temporal PCs of the lung development dataset. Each dot marks a gene, and its coordinates indicate its expression pattern over the ten developmental time points. The high dot concentrations along the periphery of the left and right hemispheres represent gene clusters whose expression levels are high early in development and decrease monotonically with time and genes whose expression levels are low early in development and increase monotonically with time, respectively. The temporal PC1 coordinate of a gene is a qualitative indicator of its lung developmental profile ( Figure 1B).

Next, mouse orthologs of genes that were up- and down-regulated in each of the four human lung cancer subtypes with respect to the NL were investigated in mouse lung development with respect to their temporal PC1 coordinates in lung development. Wilcoxon's rank sum test was used to find genes that were significantly differentially expressed between a human lung cancer subtype and the NL (see Methods). A total of 1,148 genes were significantly differentially expressed between a cancer subtype and the NL, of which 719 genes were additionally 2-fold up- or down-regulated in a lung cancer subtype ( Figure 1C).

**Figure 2:**
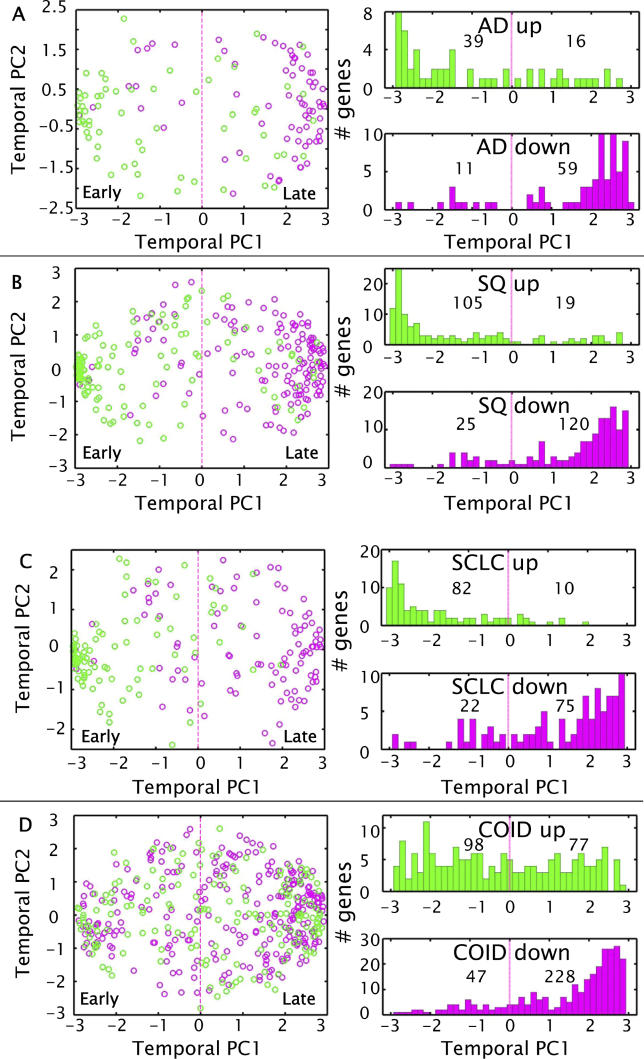
Temporal Analyses of the Significantly Differentiated Lung Cancer Genes in Murine Lung Development Analysis of mouse lung development profiles of genes 2-fold significantly up- or down-regulated in each of the four human lung cancer subtypes in relation to all 3,590 gene profiles in temporal PC1 and PC2 confirms a developmental association at the gene-by-gene scale. Shown are the mouse lung development profiles of 2-fold significantly up-regulated (green circles) and down-regulated (magenta circles) genes in human lung cancer subtypes relative to NL. (A) Up- and down-regulated genes in AD (χ ^2^ = 36.83, *p* < 0.001, OR = 13.074). (B) Up- and down-regulated genes in SQ (χ ^2^ = 119.036, *p <* 0.001, OR = 26.526). (C) Up- and down-regulated genes in SCLC (χ ^2^ = 81.584, *p <* 0.001, OR = 27.955). (D) Up- and down-regulated genes in lung COID (χ ^2^ = 72.363, *p <* 0.001, OR = 6.174).

For human lung AD, 55 genes were 2-fold significantly up-regulated and 70 were 2-fold significantly down-regulated. Figure 2A shows the temporal PC1 coordinate distribution of mouse orthologs of these 125 AD genes during lung development. The segregation of AD down-regulated genes to late mouse lung development and AD up-regulated genes to early development is statistically significant (χ ^2^ = 36.83, *p* < 0.001, odds ratio (OR) = 13.074; χ ^2^ is the Pearson χ ^2^ test with Yates continuity correction; the OR summarizes the strength of this profile segregation). The lung development profile segregation for 2-fold up- and down-regulated genes was also found in the other three lung cancers: SQ ( Figure 2B; 124 up- and 145 down-regulated genes; χ ^2^ = 119.036, *p <* 0.001, OR = 26.526), SCLC ( Figure 2C; 92 up- and 97 down-regulated genes; χ ^2^ = 81.584, *p <* 0.001, OR = 27.955), and COIDs ( Figure 2D; 175 up- and 275 down-regulated genes; χ ^2^ = 72.363, *p <* 0.001, OR = 6.174). Note that COID up-regulated genes are less strongly segregated to the early lung hemisphere than the up-regulated genes of the three other lung cancer subtypes ( Figure 2D). The premise that the set of under- or overexpressed genes might well contain biologically correlated genes that are therefore possibly statistically dependent was checked and found to be negative in the absence of a developmental segregation for under- or overexpressed cancer genes relative to a “mismatched” developing tissue background [ 19].

Temporally, murine orthologs of genes that are up-regulated in human lung cancers tend to have an expression profile that is decreasing with time during mouse lung development—though this segregation is less prominent in COIDs (comparing developmental profile ORs). Genes that are down-regulated in lung cancers tend to have a monotonically increasing expression profile during mouse lung development.

### A Mouse Lung Development Genomic Framework Discriminates between Human Lung Cancer Subtypes

The genomic level associations between human lung cancer subtypes and mouse lung development stages were examined. This is in contrast to the preceding temporal PCA–profile gene-level investigation of lung cancer and development. Each human and mouse sample was viewed as an algebraic vector of 3,590 gene features—representing its measured gene expression profile.

**Figure 3:**
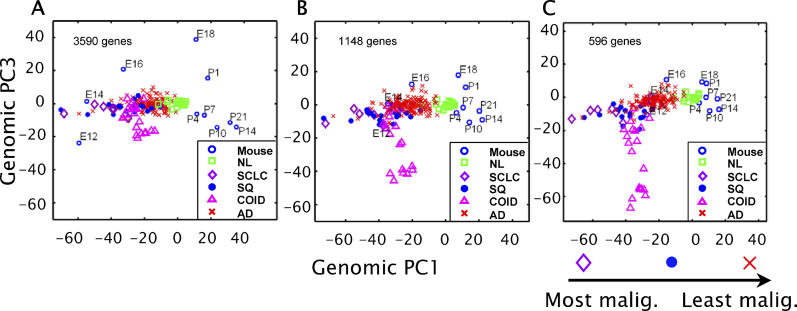
The Projection of NL and Lung Cancer Genomic Profiles onto the Genomic Mouse Lung Development Frameworks Constructed from Three Different Mouse Gene Subsets In all cases, genomic PC1 of mouse lung development is positively correlated with lung development time. Mouse lung sample placements (blue circles) are nearly contiguous as a function of their stage of development. The separation between the human lung cancer subtypes in this mouse genomic development framework is defined by a class differentiation measure (see Methods). Table S3 gives the score _pca_ values for the top-union algorithm for these gene set size parameters. Mouse, mouse lung development stages. (A) Human lung samples projected onto the mouse lung development genomic framework (genomic PC1 and PC3) of all 3,590 genes. (B) Same as (A), but for the mouse development framework constructed from the subset of 1,148 significantly differentially expressed genes in the human lung cancer subtypes. (C) Same as (B), but for the mouse development framework constructed from a further subset of 596 genes of the 1,148.

A genomic mouse lung development framework was first constructed by applying PCA to the 3,590-gene genomic axis, in contrast to the previous ten-point time axis, of the mouse lung development dataset. The PCs are now genomic, rather than temporal. Human sample profiles were then projected into this genomic mouse lung development framework ( Figure 3A). Human lung cancer samples were closer to early mouse lung development stages, while NL samples were closer to later mouse lung development stages ( Table S1). In particular, note the left to right—i.e., early to late development—placement of human sample subtypes along genomic PC1: SCLC, SQ, COID, AD, and NL (see below).

Next, the genomic mouse lung development framework was refined to improve the discrimination between human lung cancer subtypes. To do this, a new mouse lung development framework was similarly constructed based on 596 genes (a subset of the earlier set of 1,148 differentially expressed genes) that increased the separation between the human cancer subtypes ( Figure 3). A functionally annotated list of the 596 genes is included ( Dataset S1). Using this algorithm (top union; see Methods), the relationship between the cancer subtypes and developmental stage is more distinct ( Figure S5). Cancer samples now segregate even closer to the earlier mouse lung development stages, while the normal samples segregate to the late development stages. The placement of the cancer subtypes along this new genomic PC1 is unchanged from above.

Genomically, human lung cancer subtypes are more similar to earlier stages of mouse lung development, whereas the normal human lungs are more similar to the later mouse lung stages. The four cancer subtypes are further distinguishable from one another in a refined genomic mouse lung development framework. On a technical note, the top-union class discrimination methodology used here extends easily to the more general problem of feature selection for optimizing class discrimination.

### Lung COIDs Are Genomically Developmentally Distinct from the Three Other Lung Cancers

In Figure 3C, the 20 COID samples are distinguished from the other three lung cancer subtypes by their prominent variance along genomic PC3. This placement pattern suggests that COIDs are genomically distinct from the other lung cancer cancers. COID prognosis is generally better than for the other three cancer subtypes, with 5-y survival rates ranging from 27% to 75%, depending on whether the COID histological subtype is typical (better prognosis) or atypical [ 5]. Here, COID sample placement along genomic PC3 appears to be separated into two groups, possibly reflecting further histological discrimination of COIDs into typical and atypical subclasses.

### Human Lung Cancer Survival Rates Correlate with Their Genomic Placement along the Mouse Lung Development Trajectory

The left to right (early to late development) placement of the human genomic profiles of SCLC, SQ, COIDs, AD, and NL along the mouse genomic PC1 was investigated for histologic and prognostic relevance. SCLC is the most aggressive of the three lung cancer subtypes [ 5]. The 5-y survival rates for SCLC, SQ, and AD patients are 5%, 14%, and 17%, respectively [ 3]. These populational statistics suggest a link between the genomic alignment of human lung cancer on the mouse lung development trajectory and survival rate ( Table 1).

**Table 1:**
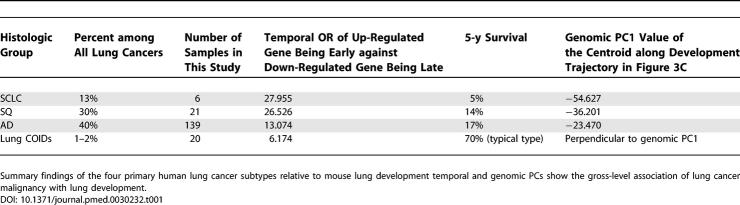
Summary of the Gross-Level Association of Lung Cancer Malignancy with Lung Development

**Figure 4:**
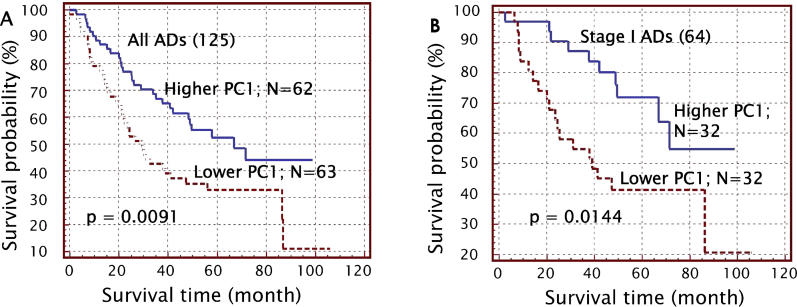
Survival Analyses of AD Patients Based on Lung Development Association Survival analyses of lung AD patients based on their mouse lung genomic PC1 coordinate show significant survival time differences between samples with early PC1 coordinates versus samples with later PC1 coordinates. The lung development genomic framework is constructed from 472 genes that are significantly up- or down-regulated in AD, SQ, or SCLC subtypes from among the set of 596 significant genes (i.e., excluding COID significant genes). (A) K-M plot for 125 AD patients separated into two quasi-equal-sized groups at the median (50th percentile) by their mouse lung genomic PC1 coordinate ( *p =* 0.0091). (B) K-M plot for 64 stage I AD patients separated into two equal-sized groups at the median by their mouse lung genomic PC1 coordinates ( *p =* 0.0144). These 64 stage I AD patient samples are selected for having more than 40% tumor cellularity, no mixed histology (adenosquamous), and patient survival information; the same criteria were described in Beer et al. [ 21].

In this human cancer dataset ( Table S2; [ 10]), clinical parameters (namely, survival time, treatment, etc.) were available for only 125 of 139 AD patients. When these 125 AD patients were ordered by their mouse genomic PC1 coordinates and split at the median into quasi-equal-sized groups, the two groups had statistically distinct survival outcomes ( Figure 4A). Furthermore, restricting the analysis to stage I AD patients and similarly dividing the population into two equal-sized groups by their mouse genomic PC1 coordinates revealed that the survival outcomes of these two groups were significantly different ( Figure 4B). The 64 stage I patient samples here were selected from the dataset with the criteria described in Beer et al. [ 21].

**Figure 5:**
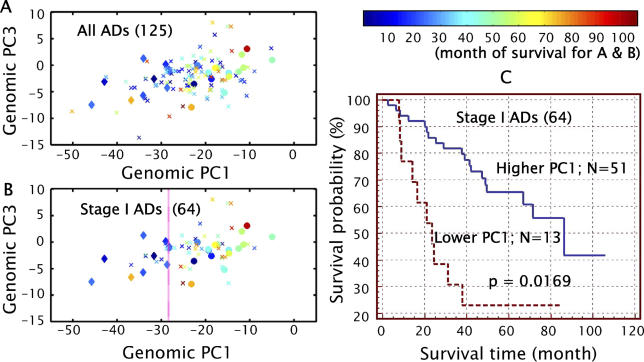
Survival Analyses of Human Lung AD Subclasses from Bhattacharjee et al. [ 10] Based on Lung Developmental Association (A) AD samples identified as forming two distinct subclasses C2 and C4 by Bhattacharjee et al. seen from the genomic PC1 and PC3 perspective of the 472-gene mouse lung development framework. Each sample point is color-coded by its survival time. Circles indicate members of class C4, diamonds indicate members of class C2, and “X”s correspond to all other AD samples. (B) Same as in (A), except that here only the stage I AD samples (such samples within C2, C4, and other) are highlighted. The mouse genomic PC1 halfway separation value between the C2 and C4 samples is noted. (C) K-M plot based on 64 stage I AD patients separated into two groups by the mouse PC1 halfway separation value from (B) ( *p =* 0.0169); the low-survival group has 13 patients and the high-survival group has 51 patients.

By hierarchically clustering the AD genomic profiles, Bhattacharjee et al. [ 10] found two distinct AD subclasses from the AD population that were significantly different in terms of their survival. The natural question in relation to the foregoing development-placement result would be where their two AD subclasses—denoted C2 and C4—localized on this mouse lung development framework. The mouse genomic PC1 and PC3 coordinates of C2 and C4 samples were plotted ( Figure 5A and 5B). With respect to the mouse lung development genomic framework, C2 and C4 remained distinct. C4 was located further right (late development) along mouse genomic PC1, while C2 was further left (early development). The foregoing result on development placement and prognosis would suggest that the C4 subclass has a better survival rate than the C2 subclass—consistent with Bhattacharjee et al.'s findings. When the mouse genomic PC1 halfway point between C2 and C4 ([maximum PC1 coordinate of C2 + minimum PC1 coordinate of C4]/2) was used to divide the overall AD patient population, the resulting two groups had even more distinctive survival rates ( Figure 5C). Cox regression was also performed with the C2–non-C2 classification combined with the corresponding grouping in genomic PC space. In the cases of both all AD samples and stage I AD samples, the developmental method gives statistically more significant *p*-values and a higher risk index ( Dataset S2).

**Figure 6:**
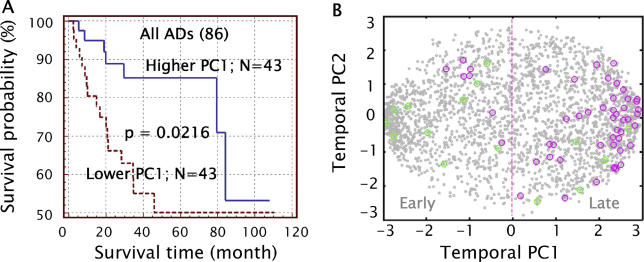
Independent Validation with Separate Human Lung AD Dataset from Beer et al. [ 21] (A) Survival analyses of 86 human lung ADs by K-M plot with patients separated into two groups by the mouse PC1 median point ( *p =* 0.0216). (B) The profiles of the 91 genes (26 cancer up-regulated [green circles] and 65 cancer down-regulated [magenta circles]) among 2,653 mouse lung developmental gene profiles.

To check if the association between AD patient survival and mouse lung development placement was particular to this lung cancer dataset, we performed a similar analysis on an independent AD dataset [ 21] relative to the mouse lung development dataset above. Patients were separated into two groups at the median of their genomic mouse PC1 coordinates. The mouse lung development framework here was constructed from 91 genes significantly differentially expressed between AD and NL. These two patient groups had significantly different survival rates ( Figure 6A). The AD up- and down-regulated 91 genes had a significantly different mouse lung development temporal profile ( Figure 6B).

**Figure 7:**
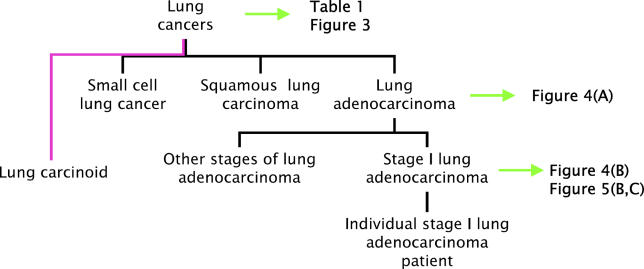
Developmental Association Predicts Human Lung Cancer Survival at Three Distinct Levels of Classical Histopathological Resolution

Together, these findings demonstrate the existence of a significant correlation between the survival outcome and genomic placement along the mouse lung development trajectory at three distinct levels of classical histopathologic resolution ( Figure 7). Patients whose lung cancer samples were associated with earlier mouse lung development stages had a poorer outcome.

### Molecular Signature for Human Lung Cancer Malignancy in the Principal Mouse Lung Development Trajectory

Genomic PC1 of mouse lung development, observed to be significantly correlated with human lung cancer survival, is a linear combination (weights) of 596 distinct gene profiles. The top 100 most heavily weighted genes in genomic PC1 were evaluated for specific function in lung cancer biology and development in the published literature (see complete list in Dataset S1). Of these, 68 genes are significantly up-regulated in human lung cancer and 32 are significantly down-regulated, with each group showing significant differences in their mouse lung development profile ( Figure 1D).

The most enriched molecular pathways in these 100 genes were pyrimidine metabolism and cell cycle. Eleven genes participate in the general cell cycle pathway (from 23 total cell-cycle-associated genes), and eight genes are involved in the pyrimidine metabolism pathway ( Table 2). De novo pyrimidine synthesis is necessary for mammalian cell proliferation. All pyrimidine metabolism and cell cycle pathways genes were up-regulated in lung cancer and most highly expressed early in lung development. In terms of general biological process, the largest categories were cell proliferation and DNA metabolism, with 26 genes each (15 shared) ( Dataset S1). Nine of the top 100 genes were involved in cell adhesion, a process generally related to metastasis ( Table 2); eight of the nine were down-regulated in lung cancer and most highly expressed late in lung development. Cell apoptosis, another general metastasis-related process was represented by six genes: *CAD, CSE1L, FXR1, NME1, PARP1,* and *TIA1.*

**Table 2:**
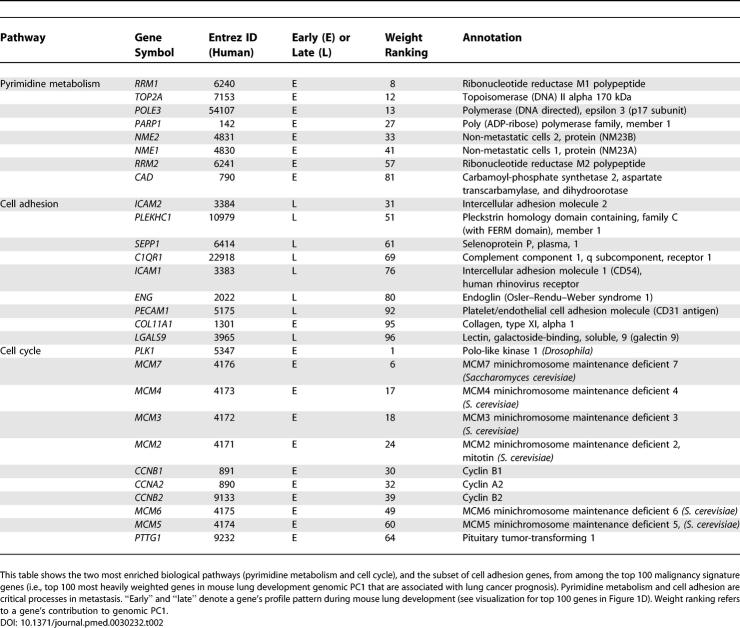
The Most Enriched Biological Pathways and Processes (Pyrimidine Metabolism, Cell Cycle, and Cell Adhesion Genes)

Of the top 100 genes, eight were directly involved in lung development, whereas 21 of the 100 have direct roles in lung cancer formation and progression ( Table 3). With regards to non-lung-specific “oncologic” association, 48 genes of the top 100 have been directly implicated in tumorigenesis, progression, or metastasis of various solid tumors. Indeed, 13 of the 20 most heavily weighted genes were cancer-related: *PLK1* (number 1 by PC1 weight ranking), *CSE1L* (number 2), *DNMT1* (number 3), *MSH6* (number 4), *MCM7* (number 6), *RRM1* (number 8), *EZH2* (number 11), *TOP2A* (number 12), *CSPG6* (number 15), *MCM4* (number 17), *MCM3* (number 18), *HSPD1* (number 19), and *PTHR1* (number 20). The group median expression value for 47 of the 100 genes increased monotonically from AD to SQ to SCLC subtypes, correlating with the relative lethality of the subtypes, whereas six genes exhibited the opposite, decreasing, group median trend: *EPHB4, GCS1, IL4R, PMP22, RARRES2,* and *TAGLN2.* Interestingly, *EPHB4* promotes cell adhesion [ 31], *RARRES2* is a receptor for retinoids that are known to inhibit growth and stimulate differentiation [ 32], and *TAGLN2* is homologous to *TAGLN,* whose expression loss marks the Ras oncogenic transformation in epithelial cells [ 33].

**Table 3:**
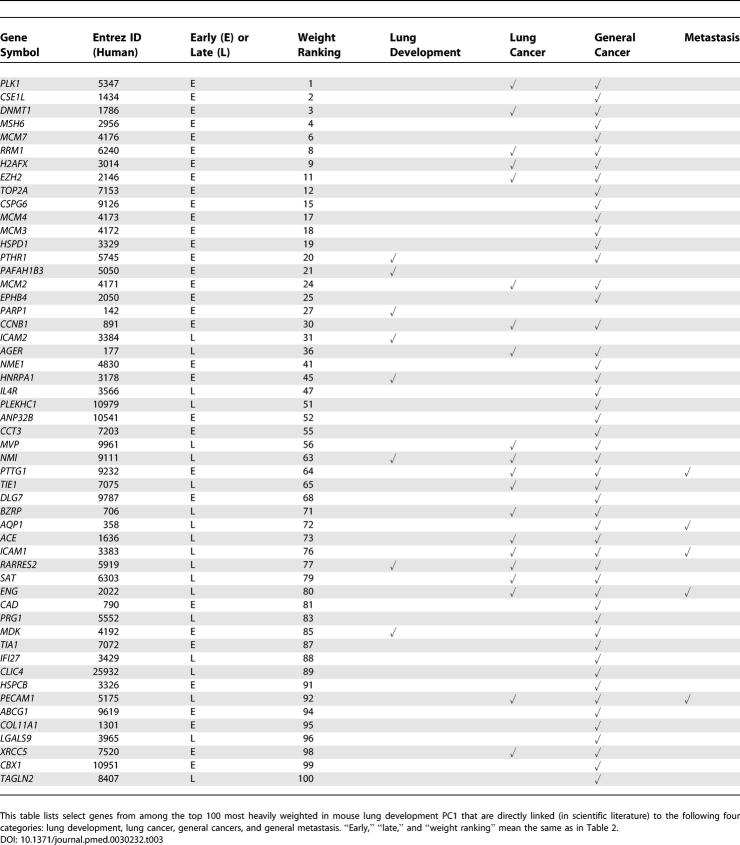
Top Genes That Are Linked to Lung Development, Lung Cancer, General Cancers, and Metastasis

Regarding metastasis, at least ten of the top 100 genes were previously reported as metastasis markers. *NME1* is a known marker for carcinoma metastasis in diverse host organs. *CCNB1* [ 34], *RRM1* [ 35], and *PTHR1* [ 36] are markers for colorectal, lung, and breast cancer metastasis, respectively. *PTTG1* is associated with lymph node metastasis of gastric carcinoma [ 37]. *PARP1* is overexpressed in malignant lymphomas [ 38]. *AGER* is associated with metastasis of pancreatic and colorectal cancers [ 39– 41]. *ENG, ICAM1,* and *PECAM1* have been linked to breast cancer metastasis [ 42, 43]. *ICAM1* is critical for the adhesion of human SCLC to endothelial cells [ 44, 45]. *AQP1* is strongly correlated with the malignancy grade in human astrocytomas [ 46]. These results suggest that the heavily weighted genes constituting lung development genomic PC1 may provide novel candidate molecules and pathways for directed investigations into the mechanisms of lung cancer biology.

## Discussion

This study demonstrates that a multi-scale modeling approach integrating the molecular dynamics of lung development provides a rational and effective framework for uncovering common developmental mechanisms of lung cancer biology and clinical outcome.

Temporally, we observed that individual genes overexpressed in human lung tumors relative to NL tissue had a significant likelihood of having corresponding mouse lung development expression profiles that decreased with time. On the other hand, genes underexpressed in lung tumors were significantly more likely to have lung development expression profiles that increased with time. A notable exception was the COID subtype: COID up-regulated genes did not have a strong tendency to have a time-decreasing lung development profile ( Figure 2D)—possibly reflecting a different cell of origin for COIDs compared with the three other lung cancer subtypes.

Genomically—with respect to a mouse lung development background—human lung cancer profiles were found to be more similar to the early stages of lung development, whereas NL profiles localized to the later stage of mouse lung development ( Figure 3). Notably, we observed a significant association between the cancer profile localization on our mouse lung development trajectory and clinical prognosis at three distinct levels of classical histopathologic resolution. At the grossest histopathologic level, the early to late development placement of group medians for the four cancer subtypes along the mouse development trajectory correspond to their group 5-y survival rates, and provides a developmental discrimination between the four classical subtypes ( Table 1). Next, significant correlations were found between the survival times of AD patients ( Figure 4A) and the placement of their samples in the same development trajectory (with the same result for the stage I subset of these patients) ( Figures 4B, 5B, S6, and S7). The earlier in development the placement of an individual human sample on the mouse lung development trajectory, the shorter the survival time was. The same development–survival relationship held in an independent AD dataset [ 21], even though that dataset had low gene-by-gene correlations [ 47] with the dataset from Bhattacharjee et al. [ 10]. In comparison with conventional histopathological methods for survival prediction, the development-based molecular method offers a new framework to investigate lung cancers. Though it might not practically or effectively replace classical histopathology in lung cancer diagnosis and prognosis in its present design, it might have greater and more fine-grained prognostic utility. For example, the Cox regression multivariate analysis using substaging, grading, and the genomic PC1 together in the case of lung AD shows that the genomic PC1 produces significant *p*-values and higher relative risk indices, while the conventional histopathology factors such as substaging and grading do not give significant *p*-values ( Figure S8; Dataset S2).

The configuration of projected human cancer genomic profiles on the cognate mouse development framework might reflect additional prognostic and phenotypic parameters. In contrast to cancer classification studies [ 10, 12, 13, 48] that apply clustering algorithms [ 49, 50] directly onto the disease datasets, the approach here does not apply PCA to the human cancer data itself. Rather, human tumor molecular profiles were projected into the PC space that was characterized by the dominant genomic structures of mouse lung development, i.e., human cancer is seen through the lens of mouse lung development. Further contrasting with other expression-based cancer survival prediction methods [ 10, 21, 51], this development-based approach involves no direct training or clustering on the disease samples themselves. In addition, the approach here does not use patient survival data for prediction: the developmental association of a disease sample is the key survival predictor. Together, these cancer–development associations suggest a developmental basis for lung cancer classification and prognosis.

Both the development expression profiles of genes up-regulated in human lung COIDs and the COID projections on mouse lung development suggest that COIDs are genomically distinct from the three other lung cancer subtypes. COID profiles were more prominently scattered along a different genomic lung development axis ( Figure 3C, PC3) from that of the other lung cancer subtypes. These observations concur with the fact that COIDs are histopathologically distinct from the other subtypes in general. A recent study by Pelosi et al. [ 8] reports that overdiagnosis of COID tumor as small cell lung carcinoma in small fragmented bronchial biopsies obtained via common fiberoptic bronchoscopy remains a significant problem. The treatment protocols for lung COIDs and small cell carcinomas are different. Thus far, hierarchical algorithms have not been able to distinguish COIDs from other lung cancer molecular profiles [ 10, 12, 13].

It is nontrivial to transition from genomic associations between phenomenological factors (e.g., development stage and disease prognosis) to functionally testable single genes or pathways, the mainstay of biological experimentation. Here, a scientific literature survey of the top 100 most heavily weighted genes constituting the prognostically relevant principal lung development trajectory showed these genes to be highly enriched for biological processes that suggest their common roles in lung development and cancer biology ( Tables 2 and 3). As shown by Kho et al. [ 19], the lung and cerebellar signatures did not cross over (the gene signature overlap between lung and central nervous system is very small), suggesting that what we were measuring was not merely proliferation, but tissue-specific development. These are genes that are not just proliferative markers but potentially developmental staging markers that can better classify patients, which in turn may be useful for prognostics and evaluating pharmacological effect. They may also provide the basis of finding novel pharmacological targets.

The idea of shared molecular mechanisms between tumorigenesis and development is an old one that originated from initial observations of common morphologic features between cancer cells and embryonic tissue [ 52]. It should be pointed out that it is not always obvious which cognate or reference development model is the best for modeling and resolving the intrinsic biology of a given tumor type. This problem largely relates to the cells of origin of a particular tumor. As has been demonstrated by numerous studies, knowledge of the basic actions of a gene or molecular pathway during development provides a rational framework for understanding its role in pathological systems such as cancer.

## Supporting Information

Dataset S1 Annotated 596-Gene Set with Extra Annotation for Top 100 Genes (514 KB XLS)

Dataset S2 Survival Analysis Report (83 KB DOC)

Figure S1 Human AD and NL samples in Murine Lung Development (A) Human lung ADs compared with NL samples in the genomic PC1 and PC2 values of mouse lung development from embryonic day 12 to postnatal day 21 in the 405 top genes' space.
(B) Human lung ADs compared with NL samples in the genomic PC1 and PC3 values of mouse lung development from embryonic day 12 to postnatal day 21 in the 405 top genes' space.
(139 KB PPT)

Figure S2 Human COID and NL Samples in Murine Lung Development (A) Human lung COIDs compared with NL samples in the genomic PC1 and PC2 values of mouse lung development from embryonic day 12 to postnatal day 21 in the 737 top genes' space.
(B) Human lung COIDs compared with NL samples in the genomic PC1 and PC3 values of mouse lung development from embryonic day 12 to postnatal day 21 in the 737 top genes' space.
(121 KB PPT)

Figure S3 Human SQ and NL Samples in Murine Lung Development (A) Human SQs compared with NL samples in the genomic PC1 and PC2 values of mouse lung development from embryonic day 12 to postnatal day 21 in the 464 top genes' space.
(B) Human SQs compared with NL samples in the genomic PC1 and PC3 values of mouse lung development from embryonic day 12 to postnatal day 21 in the 464 top genes' space.
(121 KB PPT)

Figure S4 Human SCLC and NL Samples in Murine Lung Development (A) Human SCLCs compared with NL samples in the genomic PC1 and PC2 values of mouse lung development from embryonic day 12 to postnatal day 21 in the 782 top genes' space.
(B) Human SCLCs compared with NL samples in the genomic PC1 and PC3 values of mouse lung development from embryonic day 12 to postnatal day 21 in the 782 top genes' space.
(119 KB PPT)

Figure S5 Human Cancer Samples Are Genomically Most Similar to the Earlier Mouse Lung Development Stages, whereas NL Samples Identify with the Late Development Stages (A) A stacked histogram of the mouse lung development stages closest to each human lung sample in the framework of Figure 3C, by disease subtype.
(B) The average, minimum, and maximum distances from human lung samples (in each disease subtype) to all the mouse lung development stages. The color scheme for disease subtypes follows (A). The distance measure between human samples and mouse stages is the standard Euclidean metric, calculated along the first ten genomic PCs of mouse lung development, which capture 100% of total genomic variance in the mouse lung dataset.
(74 KB PPT)

Figure S6 Survival as Surface Plot in Murine Lung Development This figure visualizes the survival time of 125 AD patients as a surface function of the genomic PC1 and PC3 in the mouse lung development genomic framework constructed from 472 genes significantly up- or down-regulated in AD, SQ, or SCLC subtypes from among the set of 596 significant genes (i.e., excluding COID significant genes). The plot is linearly color-coded, with the red spectrum representing longer survival time and the blue spectrum representing shorter survival time.
(112 KB PPT)

Figure S7 K-M Analysis of Human AD Patients in Three Developmental Groups This figure shows K-M analysis for stage I lung AD patients separated into three quasi-equal-sized groups (at the 33.33th percentile) by their mouse lung genomic PC1 coordinate ( *p* < 0.001).
(86 KB PPT)

Figure S8 K-M Analysis of Human AD Patients with Traditional Methods K-M analyses of the lung AD stage I patients (64) from Bhattacharjee et al. [ 10] by sub-staging and grades show that the traditional histopathological way of classification does not predict in fine scale the survival outcome with statistical significance.
(A) Survival analysis by sub-staging (T1 and T2).
(B) Survival analysis by grading in terms of tissue differentiation (three grades).
(138 KB PPT)

Protocol S1 Supplementary Methods (21 KB DOC)

Table S1 The Number of Samples Closest to Each Mouse Development Stage (13 KB PDF)

Table S2 The Clinical Data for the Lung Cancer Patients from Their Primary Source (207 KB XLS)

Table S3 The Scores for the Top-Union Algorithm in Action (13 KB PDF)

## Abbreviations

AD: adenocarcinoma
COID: carcinoid
K-M: Kaplan–Meier
NL: normal human lung
NSCLC: non–small cell lung cancer
OR: odds ratio
PC: principal component
PCA: principal component analysis
SCLC: small cell lung cancer
SQ: squamous cell carcinoma
