# Supplementary material for: Predicting Survival within the Lung Cancer Histopathological Hierarchy Using a Multi-Scale Genomic Model of Development
Source: PLoS Med. 2006 Jul 4;3(7):e232. doi: 10.1371/journal.pmed.0030232 (PMC1483910; doi:10.1371/journal.pmed.0030232)
Supplement: Supplementary file 1 [file pmed.0030232.st001.pdf]

The number of samples closest to each mouse development stage within each lung sample type based on 3590 homolog gene set, 1148 gene set and 596 gene set.

The # of closest samples calculated based on the 3590 human mouse homologs	E12	E14	E16	E18	P1	P4	P7	P10	P14	P21
COID	8	5	0	2	0	0	0	5	0	0
SCLC	4	2	0	0	0	0	0	0	0	0
SQ	0	14	6	0	0	1	0	0	0	0
AD	6	29	29	4	0	71	0	0	0	0
NL	0	0	1	0	2	14	0	0	0	0

The # of closest samples calculated based on the 1148 human mouse homologs	E12	E14	E16	E18	P1	P4	P7	P10	P14	P21
COID	12	6	0	0	0	0	0	2	0	0
SCLC	4	2	0	0	0	0	0	0	0	0
SQ	1	19	1	0	0	0	0	0	0	0
AD	2	92	29	2	0	14	0	0	0	0
NL	0	0	1	0	0	15	0	1	0	0

The # of closest samples calculated based on the 596 human mouse homologs	E12	E14	E16	E18	P1	P4	P7	P10	P14	P21
COID	19	1	0	0	0	0	0	0	0	0
SCLC	6	0	0	0	0	0	0	0	0	0
SQ	17	4	0	0	0	0	0	0	0	0
AD	37	86	13	0	0	3	0	0	0	0
NL	0	0	1	0	0	16	0	0	0	0
