# Supplementary material for: Predicting Survival within the Lung Cancer Histopathological Hierarchy Using a Multi-Scale Genomic Model of Development
Source: PLoS Med. 2006 Jul 4;3(7):e232. doi: 10.1371/journal.pmed.0030232 (PMC1483910; doi:10.1371/journal.pmed.0030232)
Supplement: Supplementary file 2 [file pmed.0030232.st003.pdf]

The scores for top-union algorithm in action; N is the number of top genes from each lung cancer subtype's significantly differentiated genes; ad_cn is the average distance between centroids of cancer subtypes and normal lung;· ad_cc is the average distance between centroids of the closest pairs of cancer subtypes;· t is the average tightness of cancer subtypes computed as the area of the convex hull of the human sample points divided by the number of samples in the subtype;· n_ovlp, the number of overlapping sample points between different cancer subtypes – for each pair of distinct subtypes, we count the number of samples located in the intersection of the corresponding two convex hulls.

| N | Score | ad_cn | ad_cc | t | n_ovlp |
|---|---|---|---|---|---|
| 100 | 44 | 194.66 | 47.8 | 0.95 | 172 |
| 110 | 57 | 216.93 | 53.1 | 0.73 | 148 |
| 120 | 58 | 218.54 | 54.06 | 1.37 | 142 |
| 130 | 61 | 282.91 | 82.52 | 3.2 | 90 |
| 140 | 62 | 281.89 | 83.39 | 2.46 | 91 |
| 150 | 64 | 429.11 | 123.8 | 2.32 | 140 |
| 160 | 57 | 539.63 | 161.67 | 1.2 | 167 |
| 170 | 50 | 579.14 | 170.5 | 1.32 | 178.5 |
| 180 | 55 | 588.28 | 172.39 | 1.48 | 175.5 |
| 190 | 62 | 599.51 | 179.69 | 0.86 | 176 |
| 200 | 62 | 622.99 | 188.78 | 3.23 | 154.5 |
| 210 | 84 | 675.08 | 204.22 | 2.8 | 141.5 |
| 220 | 104 | 796.71 | 244.64 | 3.64 | 137.5 |
| 230 | 103 | 879.49 | 270.39 | 4.9 | 144 |
| 240 | 79 | 701.85 | 227.77 | 4.32 | 153 |
| 250 | 89 | 736.76 | 241.7 | 3.8 | 149 |
| 260 | 86 | 760.68 | 250.34 | 6.54 | 146 |
| 270 | 83 | 754.53 | 247.6 | 6.16 | 151 |
| 280 | 89 | 774.79 | 253.11 | 6.2 | 147 |
| 290 | 54 | 649.07 | 235.85 | 6.49 | 210 |
| 300 | 46 | 639.71 | 227.84 | 7.12 | 210 |
| 310 | 41 | 646.34 | 226.03 | 8.48 | 219 |
| 320 | 52 | 683.1 | 239.88 | 8.89 | 209 |
| 330 | 44 | 637.69 | 220.38 | 8.56 | 177 |
| 340 | 40 | 598.43 | 203.05 | 7.51 | 173 |
| 350 | 52 | 579.97 | 194.76 | 5.57 | 166 |
| 360 | 59 | 632.8 | 211.38 | 5.58 | 166 |
| 370 | 55 | 658.53 | 222.57 | 5.37 | 214.5 |
| 380 | 48 | 629 | 214.01 | 5.98 | 180.5 |
| 390 | 65 | 704.97 | 235.01 | 6.1 | 177 |
| 400 | 79 | 779.66 | 260.55 | 6.98 | 170.5 |
