# Supplementary material for: Predicting Survival within the Lung Cancer Histopathological Hierarchy Using a Multi-Scale Genomic Model of Development
Source: PLoS Med. 2006 Jul 4;3(7):e232. doi: 10.1371/journal.pmed.0030232 (PMC1483910; doi:10.1371/journal.pmed.0030232)
Supplement: Supplementary file 3 [file pmed.0030232.sd002.doc]

# Kaplan-Meier analysis on the lung adenocarcinoma(AD) patients:

## All ADs, 2 equal groups, ref. Figure 4A

Survival time : Survival

Endpoint	: EndPoint

Factor codes	: Group__Use_PC1_

--------------FACTORS---------------------------------

Factor: 0	1

Sample size: 63	62

Median surv.: 66.8	29.4

SURVIVAL -----SURVIVAL PROPORTION (SE)----------------

TIME

0.1	-	0.984 (0.016)

0.5	-	-

2.6	0.984 (0.016)	-

2.8	-	0.968 (0.022)

4.1	-	0.952 (0.027)

4.2	-	0.935 (0.031)

4.5	-	0.919 (0.035)

6	-	0.903 (0.038)

6.4	0.968 (0.022)	-

6.8	0.952 (0.027)	-

7.1	0.935 (0.031)	-

7.3	-	0.887 (0.040)

7.4	-	0.871 (0.043)

7.8	-	0.855 (0.045)

8.2	0.919 (0.035)	0.823 (0.049)

8.8	-	0.806 (0.050)

9.3	-	0.790 (0.052)

9.4	0.903 (0.038)	-

10.5	0.887 (0.040)	-

12.3	0.871 (0.043)	0.774 (0.053)

13.5	-	0.758 (0.054)

13.7	-	-

14.1	-	0.742 (0.056)

14.2	-	0.726 (0.057)

15	0.855 (0.045)	0.710 (0.058)

15.2	-	0.694 (0.059)

15.3	-	-

16.5	-	0.677 (0.059)

16.6	0.838 (0.047)	-

17.3	-	-

18.7	-	0.661 (0.060)

19.7	-	0.644 (0.061)

20	0.821 (0.049)	-

20.5	0.805 (0.051)	-

21	0.788 (0.052)	0.627 (0.062)

21.1	-	0.611 (0.062)

21.6	0.771 (0.054)	-

21.9	-	0.594 (0.063)

22.3	-	0.578 (0.063)

23.4	-	0.561 (0.063)

24.3	-	0.528 (0.064)

25.1	0.754 (0.055)	-

25.3	0.738 (0.056)	-

26	0.721 (0.058)	-

26.7	-	0.512 (0.064)

29	0.704 (0.059)	-

29.3	-	-

29.4	-	0.495 (0.064)

29.6	-	0.478 (0.064)

30.1	-	0.461 (0.064)

31	-	0.444 (0.064)

31.4	-	-

32.6	-	0.427 (0.064)

33.9	0.687 (0.060)	-

34.1	-	-

35.1	0.669 (0.061)	-

35.4	-	-

37.6	0.652 (0.062)	-

37.9	-	0.409 (0.063)

38.9	-	0.391 (0.063)

40.5	-	-

40.7	0.634 (0.062)	-

41.2	-	0.373 (0.063)

41.7	-	-

41.9	0.615 (0.063)	-

42.2	-	-

43.5	-	-

44.8	-	-

45.5	-	-

46.9	-	-

47.2	-	0.353 (0.063)

48.3	0.594 (0.064)	-

48.8	0.574 (0.065)	-

49.2	-	-

49.6	0.553 (0.066)	-

50.1	-	-

50.5	-	-

52.9	-	-

54.5	-	-

56	-	-

56.1	-	0.329 (0.063)

56.3	-	-

56.7	-	-

57.6	-	-

57.8	0.525 (0.069)	-

58.5	-	-

59	-	-

59.2	-	-

59.3	-	-

60.5	-	-

61.8	-	-

62.6	-	-

66.2	-	-

66.7	-	-

66.8	0.485 (0.074)	-

71.1	-	-

71.5	0.441 (0.079)	-

72.4	-	-

74	-	-

75.4	-	-

76	-	-

76.1	-	-

76.6	-	-

78.4	-	-

79	-	-

81.9	-	-

83	-	-

84.4	-	-

85.9	-	-

86.3	-	0.219 (0.099)

86.7	-	0.110 (0.092)

88.8	-	-

91	-	-

99.1	-	-

106.1	-	-

------------------------------------------------------

Comparison of survival curves

Logrank test

Endpoint--

Observed n	29.0	42.0

Expected n	39.9	31.1

Chi-square	= 6.8053

DF	= 1

Significance	P = 0.0091

------------------------------------------------------

Hazard ratio	= 0.5394

95% CI	= 0.3337 to 0.8557

**AD stage I, 2 groups, PC1, ref. Figure 4B**

Survival time : Survival

Endpoint	: EndPoint

Select	: Select

Factor codes	: Group_PC1__StgI_

——————————————FACTORS—————————————————————————————————

Factor: 0	1

Sample size: 32	32

Median surv.: -	38.9

SURVIVAL —————SURVIVAL PROPORTION (SE)————————————————

TIME

0.5	-	-

2.6	0.969 (0.031)	-

6.4	-	0.968 (0.032)

7.8	-	0.935 (0.044)

8.2	-	0.871 (0.060)

8.8	-	0.839 (0.066)

12.3	-	0.806 (0.071)

13.7	-	-

14.2	-	0.774 (0.075)

16.5	-	0.742 (0.079)

20	-	0.710 (0.082)

21	0.936 (0.043)	-

21.1	-	0.677 (0.084)

21.6	0.904 (0.053)	-

23.4	-	0.645 (0.086)

24.3	-	0.613 (0.087)

25.3	-	0.581 (0.089)

29	0.872 (0.060)	-

31	-	0.548 (0.089)

31.4	-	-

37.6	0.838 (0.066)	-

37.9	-	0.516 (0.090)

38.9	-	0.484 (0.090)

40.5	-	-

41.2	-	0.452 (0.089)

41.7	-	-

41.9	0.802 (0.073)	-

42.2	-	-

43.5	-	-

44.8	-	-

47.2	-	0.414 (0.090)

48.8	0.762 (0.079)	-

49.2	-	-

49.6	0.719 (0.086)	-

50.1	-	-

50.5	-	-

52.9	-	-

54.5	-	-

56	-	-

56.3	-	-

57.6	-	-

58.5	-	-

59	-	-

60.5	-	-

62.6	-	-

66.8	0.640 (0.107)	-

71.1	-	-

71.5	0.548 (0.125)	-

72.4	-	-

75.4	-	-

76.1	-	-

76.6	-	-

78.4	-	-

79	-	-

81.9	-	-

83	-	-

84.4	-	-

86.3	-	0.207 (0.153)

91	-	-

99.1	-	-

106.1	-	-

————————————————————————————————————

Comparison of survival curves

Logrank test

Endpoint——

Observed n	10.0	19.0

Expected n	16.5	12.5

Chi-square	= 5.9886

DF	= 1

Significance	P = 0.0144

——————————————————————————————————————————————————————

Hazard ratio	= 0.3989

95% CI	= 0.1901 to 0.8324

**AD Stage I, 2 groups, half point of the genomic PC1 between C2 and C4 from Bhattacharjee et. al., ref. Figure 5**

Survival time : Survival

Endpoint	: EndPoint

Select	: Select

Factor codes	: Group_PC1__StgI__C2_

——————————————FACTORS—————————————————————————————————

Factor: 0	1

Sample size: 51	13

Median surv.: 86.3	23.4

SURVIVAL —————SURVIVAL PROPORTION (SE)————————————————

TIME

0.5	-	-

2.6	0.980 (0.020)	-

6.4	0.960 (0.028)	-

7.8	-	0.923 (0.074)

8.2	0.940 (0.034)	0.846 (0.100)

8.8	-	0.769 (0.117)

12.3	0.920 (0.038)	-

13.7	-	-

14.2	-	0.692 (0.128)

16.5	-	0.615 (0.135)

20	0.900 (0.043)	-

21	0.879 (0.046)	-

21.1	-	0.538 (0.138)

21.6	0.859 (0.050)	-

23.4	-	0.462 (0.138)

24.3	-	0.385 (0.135)

25.3	0.838 (0.052)	-

29	0.818 (0.055)	-

31	-	0.308 (0.128)

31.4	-	-

37.6	0.797 (0.057)	-

37.9	-	0.231 (0.117)

38.9	0.776 (0.060)	-

40.5	-	-

41.2	0.754 (0.062)	-

41.7	-	-

41.9	0.731 (0.064)	-

42.2	-	-

43.5	-	-

44.8	-	-

47.2	0.706 (0.067)	-

48.8	0.681 (0.069)	-

49.2	-	-

49.6	0.655 (0.071)	-

50.1	-	-

50.5	-	-

52.9	-	-

54.5	-	-

56	-	-

56.3	-	-

57.6	-	-

58.5	-	-

59	-	-

60.5	-	-

62.6	-	-

66.8	0.608 (0.080)	-

71.1	-	-

71.5	0.557 (0.088)	-

72.4	-	-

75.4	-	-

76.1	-	-

76.6	-	-

78.4	-	-

79	-	-

81.9	-	-

83	-	-

84.4	-	-

86.3	0.418 (0.137)	-

91	-	-

99.1	-	-

106.1	-	-

————————————————————————————————————Comparison of survival curves

Logrank test

Endpoint——

Observed n	19.0	10.0

Expected n	25.1	3.9

Chi-square	= 11.1476

DF	= 1

Significance	P = 0.0008

————————————————————————————————————

Hazard ratio	= 0.2971

95% CI	= 0.0544 to 0.4689

**AD Stage I, 3 groups, PC1, ref. Supplementary Figure 6.**

Survival time : Survival

Endpoint	: EndPoint

Select	: Select

Factor codes	: Group_PC1__StgI_3Gs_

——————————————FACTORS——————————————————————————————————————————————————

Factor: 0	1	2

Sample size: 22	21	21

Median surv.: -	66.8	37.9

SURVIVAL —————SURVIVAL PROPORTION (SE)—————————————————————————————————

TIME

0.5	-	-	-

2.6	0.955 (0.044)	-	-

6.4	-	0.950 (0.049)	-

7.8	-	-	0.952 (0.046)

8.2	-	0.900 (0.067)	0.905 (0.064)

8.8	-	-	0.857 (0.076)

12.3	-	-	0.810 (0.086)

13.7	-	-	-

14.2	-	-	0.762 (0.093)

16.5	-	-	0.714 (0.099)

20	-	0.847 (0.081)	-

21	0.909 (0.061)	-	-

21.1	-	-	0.667 (0.103)

21.6	-	0.794 (0.092)	-

23.4	-	-	0.619 (0.106)

24.3	-	-	0.571 (0.108)

25.3	-	0.741 (0.100)	-

29	-	0.688 (0.106)	-

31	-	-	0.524 (0.109)

31.4	-	-	-

37.6	0.864 (0.073)	-	-

37.9	-	-	0.476 (0.109)

38.9	-	-	0.429 (0.108)

40.5	-	-	-

41.2	-	-	0.381 (0.106)

41.7	-	-	-

41.9	0.816 (0.083)	-	-

42.2	-	-	-

43.5	-	-	-

44.8	-	-	-

47.2	-	0.619 (0.115)	-

48.8	-	0.551 (0.121)	-

49.2	-	-	-

49.6	0.757 (0.096)	-	-

50.1	-	-	-

50.5	-	-	-

52.9	-	-	-

54.5	-	-	-

56	-	-	-

56.3	-	-	-

57.6	-	-	-

58.5	-	-	-

59	-	-	-

60.5	-	-	-

62.6	-	-	-

66.8	-	0.459 (0.131)	-

71.1	-	-	-

71.5	-	0.344 (0.140)	-

72.4	-	-	-

75.4	-	-	-

76.1	-	-	-

76.6	-	-	-

78.4	-	-	-

79	-	-	-

81.9	-	-	-

83	-	-	-

84.4	-	-	-

86.3	-	-	0.190 (0.145)

91	-	-	-

99.1	-	-	-

106.1	-	-	-

———————————————————————————————————————————————————————————————————————

Comparison of survival curves

Logrank test

Endpoint——

Observed n	5.0	10.0	14.0

Expected n	12.0	8.6	8.4

Chi-square	= 8.1555

DF	= 2

Significance	P = 0.0169

**ADs, Stage I, 2 groups by substages, ref. Supplementary Figure 8A.**

Survival time : Survival

Endpoint	: EndPoint

Factor codes	: Group_StageI_substages_

--------------FACTORS---------------------------------

Factor: 0	1

Sample size: 26	38

Median surv.: 86.3	66.8

SURVIVAL -----SURVIVAL PROPORTION (SE)----------------

TIME

0.5	-	-

2.6	-	0.973 (0.027)

6.4	-	0.946 (0.037)

7.8	-	0.919 (0.045)

8.2	0.962 (0.038)	0.892 (0.051)

8.8	-	0.865 (0.056)

12.3	-	0.838 (0.061)

13.7	-	-

14.2	-	0.810 (0.065)

16.5	0.923 (0.052)	-

20	-	0.782 (0.068)

21	-	0.754 (0.071)

21.1	-	0.726 (0.074)

21.6	-	0.698 (0.076)

23.4	-	0.670 (0.078)

24.3	-	0.642 (0.080)

25.3	0.885 (0.063)	-

29	0.846 (0.071)	-

31	0.808 (0.077)	-

31.4	-	-

37.6	-	0.613 (0.081)

37.9	0.769 (0.083)	-

38.9	-	0.584 (0.082)

40.5	-	-

41.2	-	0.555 (0.083)

41.7	-	-

41.9	0.729 (0.088)	-

42.2	-	-

43.5	-	-

44.8	-	-

47.2	0.683 (0.093)	-

48.8	0.638 (0.098)	-

49.2	-	-

49.6	0.592 (0.101)	-

50.1	-	-

50.5	-	-

52.9	-	-

54.5	-	-

56	-	-

56.3	-	-

57.6	-	-

58.5	-	-

59	-	-

60.5	-	-

62.6	-	-

66.8	-	0.493 (0.094)

71.1	-	-

71.5	-	0.431 (0.101)

72.4	-	-

75.4	-	-

76.1	-	-

76.6	-	-

78.4	-	-

79	-	-

81.9	-	-

83	-	-

84.4	-	-

86.3	0.395 (0.175)	-

91	-	-

99.1	-	-

106.1	-	-

------------------------------------------------------

Comparison of survival curves

Logrank test

Endpoint--

Observed n	11.0	18.0

Expected n	13.8	15.2

Chi-square	= 1.1325

DF	= 1

Significance	P = 0.2872

------------------------------------------------------

Hazard ratio	= 0.6694

95% CI	= 0.3220 to 1.3988

**AD Stage I, 3 groups by differentiation grades, ref. Supplementary Figure 8B**

Survival time : Survival

Endpoint	: EndPoint

Factor codes	: Group_DifferentiationInStageI_

--------------FACTORS--------------------------------------------------

Factor: 0	1	2

Sample size: 23	19	22

Median surv.: 71.5	-	48.8

SURVIVAL -----SURVIVAL PROPORTION (SE)---------------------------------

TIME

0.5	-	-	-

2.6	-	0.944 (0.054)	-

6.4	-	-	0.955 (0.044)

7.8	-	-	0.909 (0.061)

8.2	-	-	0.818 (0.082)

8.8	-	-	0.773 (0.089)

12.3	-	-	0.727 (0.095)

13.7	-	-	-

14.2	-	-	0.682 (0.099)

16.5	-	0.889 (0.074)	-

20	0.955 (0.044)	-	-

21	-	0.833 (0.088)	-

21.1	-	0.778 (0.098)	-

21.6	0.909 (0.061)	-	-

23.4	-	-	0.636 (0.103)

24.3	0.864 (0.073)	-	-

25.3	-	-	0.591 (0.105)

29	0.818 (0.082)	-	-

31	-	0.722 (0.106)	-

31.4	-	-	-

37.6	0.773 (0.089)	-	-

37.9	-	0.662 (0.113)	-

38.9	0.727 (0.095)	-	-

40.5	-	-	-

41.2	-	-	0.542 (0.107)

41.7	-	-	-

41.9	0.679 (0.100)	-	-

42.2	-	-	-

43.5	-	-	-

44.8	-	-	-

47.2	-	0.588 (0.122)	-

48.8	-	-	0.492 (0.108)

49.2	-	-	-

49.6	-	0.515 (0.127)	-

50.1	-	-	-

50.5	-	-	-

52.9	-	-	-

54.5	-	-	-

56	-	-	-

56.3	-	-	-

57.6	-	-	-

58.5	-	-	-

59	-	-	-

60.5	-	-	-

62.6	-	-	-

66.8	0.566 (0.133)	-	-

71.1	-	-	-

71.5	0.453 (0.147)	-	-

72.4	-	-	-

75.4	-	-	-

76.1	-	-	-

76.6	-	-	-

78.4	-	-	-

79	-	-	-

81.9	-	-	-

83	-	-	-

84.4	-	-	-

86.3	-	-	0.246 (0.182)

91	-	-	-

99.1	-	-	-

106.1	-	-	-

-----------------------------------------------------------------------

Comparison of survival curves

Logrank test

Endpoint--

Observed n	9.0	8.0	12.0

Expected n	11.4	8.2	9.4

Chi-square	= 1.2095

DF	= 2

Significance	P = 0.5462

**Survival analyses of 86 human lung adenocarcinoma data from Beer et.al., 2 groups by the mouse PC1 median point. Ref. Figure 7.**

Survival time : Followup_time__months_

Endpoint	: death__1_dead_0_alive_

Factor codes	: Group__median__PC1_

--------------FACTORS---------------------------------

Factor: 0	1

Sample size: 43	43

Median surv.: -	-

SURVIVAL -----SURVIVAL PROPORTION (SE)----------------

TIME

1.5	-	-

1.6	-	-

2.4	-	0.976 (0.024)

2.6	-	0.952 (0.033)

4	-	0.929 (0.040)

4.5	-	-

4.9	-	-

5.4	0.975 (0.025)	-

5.8	-	0.905 (0.045)

6.1	-	-

7.2	-	0.881 (0.050)

8.3	-	-

8.4	0.949 (0.035)	-

8.5	-	-

8.7	-	0.856 (0.055)

9.6	-	0.831 (0.058)

10	-	-

10.1	-	0.805 (0.062)

10.4	-	-

12.2	-	-

12.5	-	-

13	-	-

14.5	-	-

14.9	-	0.777 (0.066)

15.1	-	-

16.6	-	0.749 (0.069)

17.7	-	-

19	0.920 (0.045)	-

19.5	-	-

19.9	0.889 (0.053)	-

20.1	-	0.720 (0.072)

20.2	-	-

20.6	-	0.692 (0.075)

21.1	-	-

21.2	-	0.661 (0.077)

24.4	-	-

25.2	-	-

25.3	-	-

26.8	-	-

28.2	-	0.630 (0.080)

28.3	-	-

29.4	-	-

29.6	0.852 (0.062)	-

30.6	-	-

30.8	-	-

32.2	-	-

34.1	-	-

34.2	-	-

34.6	-	0.591 (0.084)

34.7	-	0.551 (0.087)

36	-	-

36.5	-	-

39.1	-	-

40	-	-

43.8	-	-

45.8	-	0.501 (0.093)

47	-	-

48.1	-	-

48.2	-	-

52.4	-	-

52.9	-	-

54.6	-	-

54.8	-	-

60.5	-	-

61.8	-	-

63.4	-	-

65.4	-	-

68.1	-	-

70.7	-	-

77.8	-	-

78.5	-	-

79.5	0.710 (0.140)	-

82.4	-	-

83.7	-	-

84.1	0.532 (0.186)	-

85.2	-	-

87.7	-	-

91.8	-	-

91.9	-	-

93.7	-	-

98.7	-	-

107.9	-	-

108.2	-	-

110.6	-	-

------------------------------------------------------

Comparison of survival curves

Logrank test

Endpoint--

Observed n	7.0	17.0

Expected n	12.6	11.4

Chi-square	= 5.2784

DF	= 1

Significance	P = 0.0216

------------------------------------------------------

Hazard ratio	= 0.3724

95% CI	= 0.1744 to 0.8707

Cox proportional-hazard regression tests on lung adenocarcinoma patients:

Survival time : Survival

Endpoint	: EndPoint

Method	: Enter

Sample size	= 64

--- Overall Model Fit --------------------------------------------------

Null model -2 Log Likelihood = 214.41596

Full model -2 Log Likelihood = 207.24696

Chi-square = 7.1690; DF = 3; P = 0.0667

--- Coefficients and Standard Errors -----------------------------------

Covariate	b	SE	P	95% CI	Exp(b)

**Group_PC1**_	0.9127	0.4266	**0.0324**	0.0808 to 1.7445	**2.4909**

Group_Diff	0.0267	0.2359	0.9100	-0.4333 to 0.4866	1.0270

Group_Stag	0.4270	0.3901	0.2737	-0.3337 to 1.1876	1.5326

Legend:

Group_PC1_ : grouping of the stage I patients into 2 using median value of genomic PC1

Group_Diff : grouping of the stage I patients into 3 using the three differentiation grades

Group_Stag : grouping of the stage I patients into 2 using the substaging (T1 and T2) in histopathology

Survival time : Survival

Endpoint	: EndPoint

Method	: Enter

Sample size	= 64

--- Overall Model Fit --------------------------------------------------

Null model -2 Log Likelihood = 214.41596

Full model -2 Log Likelihood = 207.25975

Chi-square = 7.1562; DF = 2; P = 0.0279

--- Coefficients and Standard Errors -----------------------------------

Covariate	b	SE	P	95% CI	Exp(b)

**Group_PC1**_	0.9316	0.3920	**0.0175**	0.1672 to 1.6960	**2.5386**

Group_Stag	0.4233	0.3888	0.2763	-0.3348 to 1.1814	1.5270

Legend:

Group_PC1_ : grouping of the stage I patients into 2 using median value of genomic PC1

Group_Stag : grouping of the stage I patients into 2 using the substaging (T1 and T2) in histopathology

Survival time : Survival

Endpoint	: EndPoint

Method	: Enter

Sample size	= 64

--- Overall Model Fit --------------------------------------------------

Null model -2 Log Likelihood = 214.41596

Full model -2 Log Likelihood = 206.25830

Chi-square = 8.1577; DF = 2; P = 0.0169

--- Coefficients and Standard Errors -----------------------------------

Covariate	b	SE	P	95% CI	Exp(b)

**Group_PC1**_	0.6721	0.2649	**0.0112**	0.1556 to 1.1886	**1.9583**

Group_Diff	-0.0425	0.2428	0.8609	-0.5160 to 0.4309	0.9583

Legend:

Group_PC1_ : grouping of the stage I patients into 3 equal-sized groups using the value of genomic PC1

Group_Diff : grouping of the stage I patients into 3 using the three differentiation grades

Survival time : Survival

Endpoint	: EndPoint

Method	: Enter

Sample size	= 64

--- Overall Model Fit --------------------------------------------------

Null model -2 Log Likelihood = 214.41596

Full model -2 Log Likelihood = 205.98958

Chi-square = 8.4264; DF = 2; P = 0.0148

--- Coefficients and Standard Errors -----------------------------------

Covariate	b	SE	P	95% CI	Exp(b)

C2_NonC2	0.0354	0.6931	0.9592	-1.3161 to 1.3870	1.0361

**PC1__C2___**	1.2457	0.4514	**0.0058**	0.3655 to 2.1259	**3.4754**

Legend:

C2_NonC2	: grouping of the stage I patients into 2 using C2 and Non C2 classes from Bhattacharjee et. al.

PC1__C2___ : grouping of the stage I patients into 2 using the mouse genomic PC1 halfway point between C2 and C4.

C2 and C4 are classes from Bhattacharjee et. al.

Survival time : Survival

Endpoint	: EndPoint

Method	: Enter

Sample size	= 125

--- Overall Model Fit --------------------------------------------------

Null model -2 Log Likelihood = 611.93071

Full model -2 Log Likelihood = 603.20985

Chi-square = 8.7209; DF = 2; P = 0.0128

--- Coefficients and Standard Errors -----------------------------------

Covariate	b	SE	P	95% CI	Exp(b)

C2_NonC2	-0.6134	0.4331	0.1566	-1.4579 to 0.2310	0.5415

**PC1__C2___**	0.5273	0.2844	**0.0638**	-0.0274 to 1.0819	**1.6943**

Legend:

C2_NonC2	: grouping of the patients into 2 using C2 and Non C2 classes from Bhattacharjee et. al.

PC1__C2___ : grouping of the patients into 2 using the mouse genomic PC1 halfway point between C2 and C4.

C2 and C4 are classes from Bhattacharjee et. al.
