# Supplementary material for: Predicting Survival within the Lung Cancer Histopathological Hierarchy Using a Multi-Scale Genomic Model of Development
Source: PLoS Med. 2006 Jul 4;3(7):e232. doi: 10.1371/journal.pmed.0030232 (PMC1483910; doi:10.1371/journal.pmed.0030232)
Supplement: Supplementary file 4 [file pmed.0030232.sd003.doc]

**A)** Based on the 1,148 top genes, we seek a gene subset that maximizes score_pca_. We follow a selection procedure that: 1) picks the first N top genes from each of the four p-valued ordered list of genes differentially-expressed between the cancer subtypes and normal lung; 2) constructs the corresponding genomic mouse lung development framework; 3) projects all human samples into this framework; and 4) computes the corresponding score_pca_(N). Iterating for N, we find the maximal score_pca_ for N=220 – the union of which are 596 unique genes.

The following pseudo-code details this top-union algorithm:

1. Start with the 1,148-gene dataset – the union of four Wilcoxon p-value (Bonferroni corrected) ordered list of genes differentially-expressed between each cancer subtype and the normal lung;
2. Define N as the threshold for selecting the first N genes from each ordered cancer subtype list;
3. For each N and the resulting combined (union) gene set ;
4. Do genomic PCA of mouse lung development, project human samples, and compute the score_pca_(N);
5. End
6. Pick N which produces maximal score_pca_(N).

**B)** Supplementary notes for the projection of human samples:

Φ_matrix of PC's_ is the orthonomal matrix whose column vectors are PC’s.

x_old_vector_ , x_new_vector_ = expression profiles of a sample.

The projection of human samples into mouse development is done as follows:

1) Normalize the human tumor samples with respect to the normal human lung samples by subtracting the tumors with normal signals and dividing by the normal variance.
2) Applying the same transformation using matrix Φ'_matrix_ on human samples.
